# Uncovering Zn^2+^ as a cofactor of FAD-dependent *Pseudomonas aeruginosa* PAO1 d-2-hydroxyglutarate dehydrogenase

**DOI:** 10.1016/j.jbc.2023.103007

**Published:** 2023-02-11

**Authors:** Joanna A. Quaye, Giovanni Gadda

**Affiliations:** 1 Department of Chemistry, Georgia State University, Atlanta, Georgia, USA; 2 Department of Biology, Georgia State University, Atlanta, Georgia, USA; 3 The Center for Diagnostics and Therapeutics, Georgia State University, Atlanta, Georgia, USA

**Keywords:** *Pseudomonas aeruginosa*d-2-hydroxyglutarate dehydrogenase, d-2-hydroxyglutarate, d-malate, reductive-half reaction, Zn^2+^, metallo flavoprotein, flavin, inductively coupled plasma mass spectrometry, induced-fit rapid-rearrangement mechanism, D2HG, d-2-hydroxyglutarate, D2HGDH, d-2-hydroxyglutarate dehydrogenase, ICP-MS, inductively coupled plasma-mass spectrometry, LB, Luria–Bertani, LDH, lactate dehydrogenase, *Pa*D2HGDH, D2HGDH from *Pseudomonas aeruginosa*, PMS, phenazine methosulfate

## Abstract

*Pseudomonas aeruginosa* couples the oxidation of d-2-hydroxyglutarate (D2HG) to l-serine biosynthesis for survival, using d-2-hydroxyglutarate dehydrogenase from *P. aeruginosa* (*Pa*D2HGDH). Knockout of *Pa*D2HGDH impedes *P. aeruginosa* growth, making *Pa*D2HGDH a potential target for therapeutics. Previous studies showed that the enzyme's activity increased with Zn^2+^, Co^2+^, or Mn^2+^ but did not establish the enzyme's metal composition and whether the metal is an activator or a required cofactor for the enzyme, which we addressed in this study. Comparable to the human enzyme, *Pa*D2HGDH showed only 15% flavin reduction with D2HG or d-malate. Upon purifying *Pa*D2HGDH with 1 mM Zn^2+^, the Zn^2+^:protein stoichiometry was 2:1, yielding an enzyme with ∼40 s^−1^*k*_cat_ for d-malate. Treatment with 1 mM EDTA decreased the Zn^2+^:protein ratio to 1:1 without changing the kinetic parameters with d-malate. We observed complete enzyme inactivation for the metalloapoenzyme with 100 mM EDTA treatment, suggesting that Zn^2+^ is essential for *Pa*D2HGDH activity. The presence of Zn^2+^ increased the flavin N^3^ atom p*K*_a_ value to 11.9, decreased the flavin ε_450_ at pH 7.4 from 13.5 to 11.8 mM^−1^ cm^−1^, and yielded a charged transfer complex with a broad absorbance band >550 nm, consistent with a Zn^2+^-hydrate species altering the electronic properties of the enzyme-bound FAD. The exogenous addition of Zn^2+^, Co^2+^, Cd^2+^, Mn^2+^, or Ni^2+^ to the metalloapoenzyme reactivated the enzyme in a sigmoidal pattern, consistent with an induced fit rapid-rearrangement mechanism. Collectively, our data demonstrate that *Pa*D2HGDH is a Zn^2+^-dependent metallo flavoprotein, which requires Zn^2+^ as an essential cofactor for enzyme activity.

Metals are an essential class of elements necessary for the survival of all life forms, including bacteria, plants, and humans (1, 2, 3, 4, 5, 6, 7, 8, 9, 10, 11, 12). The various properties of metals make them vital for use in many disciplines, with applications in cellular metabolism, medicine, pharmaceutics, engineering, energy, transport, finance, and security (11, 13, 14, 15, 16, 17, 18). Metal ions play a significant role in biological systems by altering vital metabolic processes for life (10, 17, 19). They regulate cellular osmotic pressure and electrical charges, control immunity, serve as antioxidants and pro-oxidants and antimicrobial agents, and actively participate in signal transduction in response to stimuli (4, 6, 7, 8, 9, 10, 11, 17, 19). Metal deficiencies lead to impaired growth, development retardation, metabolic malfunction, carcinogenesis, or death (1, 2, 9, 10). The metal magnesium is required in cells for DNA stability (5). Proteins like hemoglobin, which uses iron to transport dioxygen (O_2_) throughout the body (7, 8), require metals to function.

A metal-containing protein is called a metalloprotein (20). Most metalloproteins exploit metals as cofactors because of their stability in multiple oxidation states and ability to form strong kinetically favored bonds. Other metalloproteins use metals for structural support (20, 21, 22). Carbonic anhydrase was one of the first enzymes discovered to require a metal for catalysis (23). Afterward, several metal-dependent enzymes were identified, including the Zn^2+^-containing carboxypeptidase A (24). Some flavin-containing enzymes (flavoproteins) contain metals (25, 26, 27, 28, 29, 30). This metal–flavin combination gives rise to the metallo flavoprotein class of enzymes. Studies on flavin-dependent *Methanobacterium formicicum* formate dehydrogenase, a mammalian histone demethylase, d-lactate dehydrogenase (LDH), avian sulfhydryl oxidase, and mammalian d-2-hydroxyglutarate dehydrogenases (D2HGDHs) have demonstrated activation or inhibition by metals including Zn^2+^, Ni^2+^, Co^2+^, Fe^2+^, Mn^2+^, Cd^2+^, and Fe^3+^ (25, 26, 27, 28, 29, 30, 31, 32, 33, 34, 35, 36, 37, 38).

Different metals have different effects and functions in enzymes. Reports suggest that metals may be involved in substrate binding in the flavin-dependent α-hydroxy acid–oxidizing enzymes, whereas others suggest roles in flavin reduction (27, 39, 40, 41). During enzyme turnover, the bound flavin undergoes a reduction–oxidation cycle, in which the flavin is reduced during the first half of the reaction (39). For flavoproteins that experience a one-electron reduction yielding highly reactive semiquinone radicals, electrons are typically transferred to oxidized cytochromes and metals like Fe^3+^ for flavin reoxidation (39). In a study on mitochondrial 2-oxoglutarate oxygenase, inhibition of the glutathione-mediated 2-hydroxyglutarate oxidation is observed when the essential Fe^2+^ or Fe^3+^ is substituted with Zn^2+^, Ni^2+^, Cu^2+^, Mn^2+^, or Co^2+^ (32). Zn^2+^ and Co^2+^ increase the activity of D2HGDH from rat liver and d-LDHs from different species (26, 27, 30); however, they inhibit chromate reductase activity (26). Although Mg^2+^ is mainly considered a neutral metal or an inhibitory metal, it has been observed to activate yeast hydroxy acid dehydrogenase and d-lactate-cytochrome (27, 41, 42). Mn^2+^, on the other hand, can function as an activator or an inhibitor (26, 30, 32, 33). Native-bound metals can be removed with EDTA to render an enzyme inactive and functionally replaced by exogenous metal addition (27, 41, 42), as is the case for mitochondrial hydroxy acid dehydrogenase (27). The FAD-dependent d-LDH from yeast and *Medasphora elsdenii* has been reported to contain an essential Zn^2+^ per FAD (43). Yet, no mechanisms have been identified for these metals' activation or inhibitory processes in metallo flavoenzymes, including D2HGDH.

D2HGDH from *Pseudomonas aeruginosa* PAO1 (*Pa*D2HGDH) has become a flavoprotein of recent interest following its identification as a potential therapeutic target against *P. aeruginosa* (44). *P. aeruginosa* is an opportunistic multidrug-resistant bacterium (45, 46, 47), which causes fatal nosocomial infections in humans (48, 49). *Pa*D2HGDH plays a vital role in *P. aeruginosa* survival, with no compensatory activity after D2HGDH gene knockouts (31, 50). The enzyme is an FAD-dependent dehydrogenase, which does not react with molecular oxygen, follows ping–pong bi–bi steady-state kinetics (45), and oxidizes D2HG and d-malate as substrates (30, 31, 44). *Pa*D2HGDH, like the FAD-dependent LDH, FAD-dependent glycolate oxidase, and human D2HGDH belongs to the FAD-dependent α-hydroxy acid–oxidizing enzyme class of proteins that have been demonstrated to use metals or other coenzymes such as NAD^+^ for maximum catalysis (39). Despite the suggestion of *Pa*D2HGDH as a metallo flavoprotein, there is no understanding of its metal composition, binding and coordination, significance and importance, and activation mechanism.

In this study, His-tagged *Pa*D2HGDH from *P. aeruginosa* PAO1 has been recombinantly expressed, purified to high levels, and investigated for its activity in the presence of Zn^2+^. Zn^2+^ has been uncovered as an essential cofactor for *Pa*D2HGDH, rather than an enzyme activator, identifying the enzyme as a metallo flavoprotein. The kinetic and inductively coupled plasma-mass spectrometry (ICP-MS) analysis data of *Pa*D2HGDH treated with and without EDTA are discussed. This study proposes a mechanism for metal reactivation of inactive *Pa*D2HGDH with potential protein ligands for metal binding in *Pa*D2HGDH.

## Results

### Substrate-induced reduction of *Pa*D2HGDH as purified without Zn^2+^

**Figure 1:**
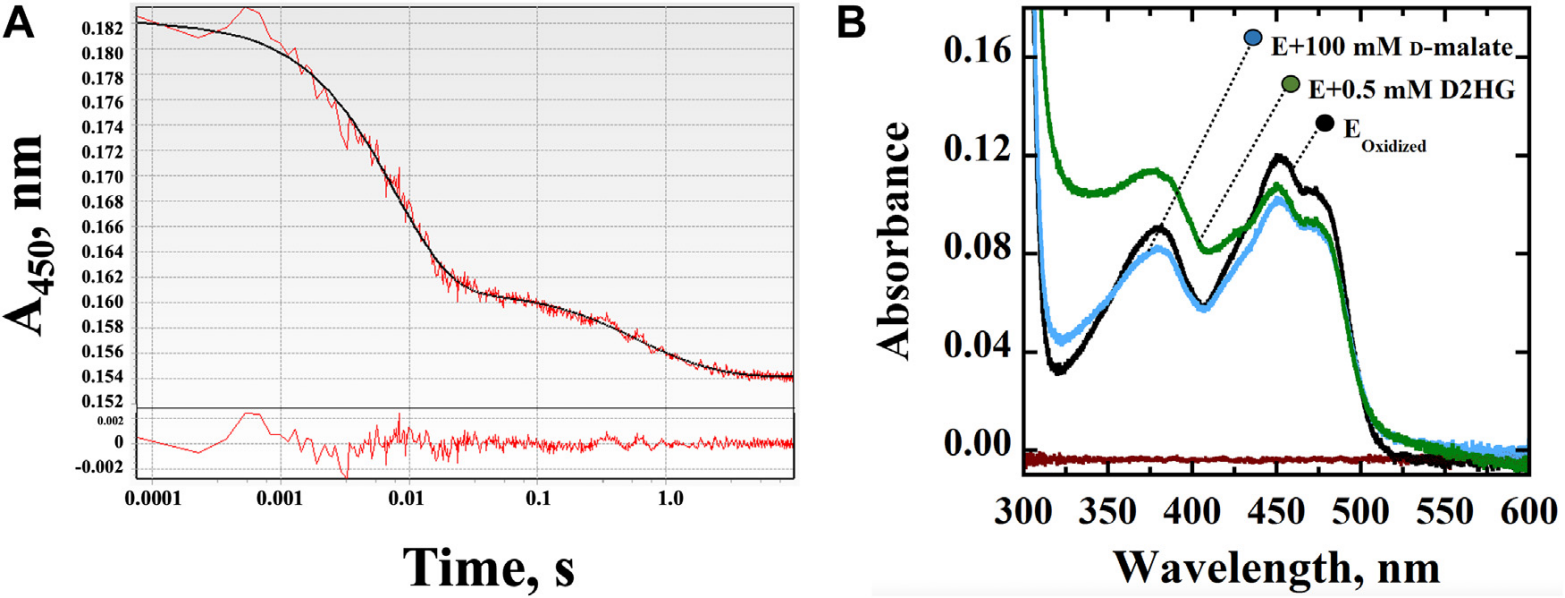
**Flavin reduction of *Pa*D2HGDH.***Left panel*, a stopped-flow trace of the reductive-half reaction of *Pa*D2HGDH showing incomplete flavin reduction of *Pa*D2HGDH with 40 mM d-malate, fit to Equation 3. Note the log time scale. The instrument dead time following the mixing of the enzyme with the substrate was 2.2 ms. The residuals of the data fitting are shown in the *bottom panel*. *Right panel*, absorption spectra of *Pa*D2HGDH showing incomplete flavin reduction with saturating concentrations of d-malate at 100 mM or D2HG at 0.5 mM. The assay was carried out in 25 mM Tris–Cl, pH 7.4, at 25 °C. D2HG, d-2-hydroxyglutarate; *Pa*D2HGDH, D2HGDH from *Pseudomonas aeruginosa*.

To characterize *Pa*D2HGDH as purified in its kinetic properties, the reductive-half reaction of the enzyme purified in the absence of ZnCl_2_ (*vide infra*) in the purification buffers was investigated by following the substrate-induced absorbance changes of the enzyme-bound flavin at 450 nm. Time-resolved absorption spectroscopy at varying concentrations of d-malate showed incomplete flavin reduction, that is, ∼15%, in two reaction phases (Fig. 1) and irrespective of the substrate concentration used. Similar results were obtained with D2HG (Fig. 1). These data are consistent with the data previously reported for the human enzyme, for which no explanation was provided to account for the partial flavin reduction (51).

### Effect of metals on *Pa*D2HGDH activity

Previous studies on mammalian D2HGDHs showed that the enzyme activity could be enhanced in the presence of divalent metals (25, 26, 27, 28, 29, 30, 31, 32, 33, 34, 35, 36, 37, 38). Thus, divalent metals were investigated for their effects on *Pa*D2HGDH activity. Enzyme activity was measured with 5 mM d-malate and 1 mM phenazine methosulfate (PMS) at pH 7.4 in the presence of 0.1 mM chloride salts of Co^2+^, Ca^2+^, Zn^2+^, Mn^2+^, Mg^2+^, Fe^2+^, Cd^2+^, Cu^2+^, or Ni^2+^. As shown in Table 1, Co^2+^, Zn^2+^, Mn^2+^, Ni^2+^, and Cd^2+^ increased the enzyme activity; in contrast, Ca^2+^, Cu^2+^, and Mg^2+^ did not affect the enzyme's activity.

**Table 1:** Activity assay of *Pa*D2HGDH after the exogenous addition of metals

Rate, s^−1^	E	E + Co^2+^	E + Zn^2+^	E + Mn^2+^	E + Ni^2+^	E + Cd^2+^	E + Ca^2+^	E + Cu^2+^	E + Mg^2+^

0.2	11	7	2	9	5	0.2	0.2	0.2

Activity assays were carried out with 100 μM metal chloride salts, 5 mM d-malate, and 1 mM PMS. ≤5% standard error was recorded for all numbers obtained from the average of the experiment done in triplicates.

Abbreviation: E, *Pa*D2HGDH.

### ICP-MS analysis of *Pa*D2HGDH

ICP-MS was carried out to determine whether metals were bound to the enzyme as purified after recombinant expression in *Escherichia coli*. The data revealed significant, although nonstoichiometric, amounts of Mg^2+^ and Zn^2+^ bound to the enzyme (Table 2). In contrast, other metals like Co^2+^, Mn^2+^, Fe^2+^, Cd^2+^, and Ni^2+^ were present only in trace amounts. Thus, Zn^2+^ was the only metal present in significant quantities in the recombinantly expressed *Pa*D2HGDH that also yielded an increase in enzyme catalytic activity. Subsequent preparations of *Pa*D2HGDH were carried out in the presence of 1 mM ZnCl_2_ in all purification buffers to evaluate whether the Zn^2+^:protein stoichiometry could be increased. When the ICP-MS analysis was performed on the enzyme prepared in the presence of ZnCl_2_, labeled E-Zn^2+^, the mol Zn^2+^:mol protein ratio was ∼2.0, whereas that of Mg^2+^ was ∼0.2 (Table 2).

**Table 2:** Metal composition of the different forms of *Pa*D2HGDH

Metal (M)	[M]_E_, μM (mol M:mol E)^a^	[M]_E-Zn_^2+^, μM (mol M:mol E)^b^	[M]_E-Zn_^2+^_1 mM EDTA_, μM (mol M:mol E)^c^	[M]_E-Zn_^2+^_100 mM EDTA_, μM (mol M:mol E)^d^
Zn^2+^	25 (0.1:1)	420 (2.2:1)	250 (1.2:1)	47 (0.2:1)
Mg^2+^	112 (0.4:1)	39 (0.2:1)	28 (0.1:1)	146 (0.5:1)

Abbreviations: E, *Pa*D2HGDH; M, metal.

a 288 μM protein.

b 195 μM protein.

c 210 μM protein.

d 272 μM protein. <7% standard error was recorded for all numbers. Cd^2+^, Ni^2+^, Co^2+^, Mn^2+^, and Fe^2+^ were found in trace amounts of <0.2%.

The E-Zn^2+^ enzyme was then treated with either 1 mM EDTA or 100 mM EDTA to explore whether Zn^2+^ was labile and could be removed from the enzyme. A molar Zn^2+^:protein stoichiometry of ∼1.0 was determined using ICP-MS for the enzyme mildly treated with EDTA, labeled E-Zn^2+^_1 mM EDTA,_ and of ∼0 for the enzyme harshly treated with EDTA, labeled E-Zn^2+^_100 mM EDTA_. Apart from Mg^2+^, all other metals were identified in trace amounts, irrespective of the EDTA treatment (Table 2).

### Steady-state kinetics of *Pa*D2HGDH with various Zn^2+^:protein stoichiometries

To investigate the effects of Zn^2+^ loading on *Pa*D2HGDH activity, the steady-state kinetic parameters with d-malate and 1 mM PMS as substrates for E-Zn^2+^, E-Zn^2+^_1 mM EDTA,_ and E-Zn^2+^_100 mM EDTA_ were measured with the method of the initial rates at pH 7.4 and 25 °C. The data showed a Michaelis–Menten pattern with both E-Zn^2+^ and E-Zn^2+^_1 mM EDTA_, allowing for the determination of the apparent *k*_cat_, *K*_*m*_, and *k*_cat_/*K*_*m*_ values. As shown in Table 3, the kinetic parameters for E-Zn^2+^ and E-Zn^2+^_1 mM EDTA_ were comparable. No enzymatic activity was determined for E-Zn^2+^_100 mM EDTA,_ irrespective of the substrate concentration.

**Table 3:** Kinetic properties of the different forms of *Pa*D2HGDH^a^

Kinetic parameters	E-Zn^2+^	E-Zn^2+^_1 mM EDTA_	E-Zn^2+^_100 mM EDTA_
*k*_cat_, s^−1^	38 ± 1	39 ± 1	NA
*K*_*m*_, mM	5.1 ± 0.4	4.0 ± 0.4	NA
*k*_cat_/*K*_*m*_ M^−1^s^−1^	7000 ± 500	10,000 ± 1000	NA

Abbbreviations: E, *Pa*D2HGDH; NA, no enzyme activity detected.

a Enzyme activity was measured at varying concentrations of d-malate and 1 mM PMS, in 25 mM NaPO_4_, pH 7.4, at 25 °C.

To investigate the effect of Zn^2+^ loading on the ability of *Pa*D2HGDH to utilize molecular oxygen as an electron acceptor, E-Zn^2+^ was tested for its reactivity with O_2_ by mixing the enzyme with various concentrations of d-malate without PMS in a Clark-type oxygen electrode at pH 7.4 and 25 °C. There was no oxygen consumption under these conditions, consistent with previous data on the enzyme purified without exogenous Zn^2+^ (44). Thus, *Pa*D2HGDH is a dehydrogenase lacking the ability to use molecular oxygen as an electron acceptor irrespective of the presence of Zn^2+^.

### Substrate-induced reduction of E-Zn^2+^

**Figure 2:**
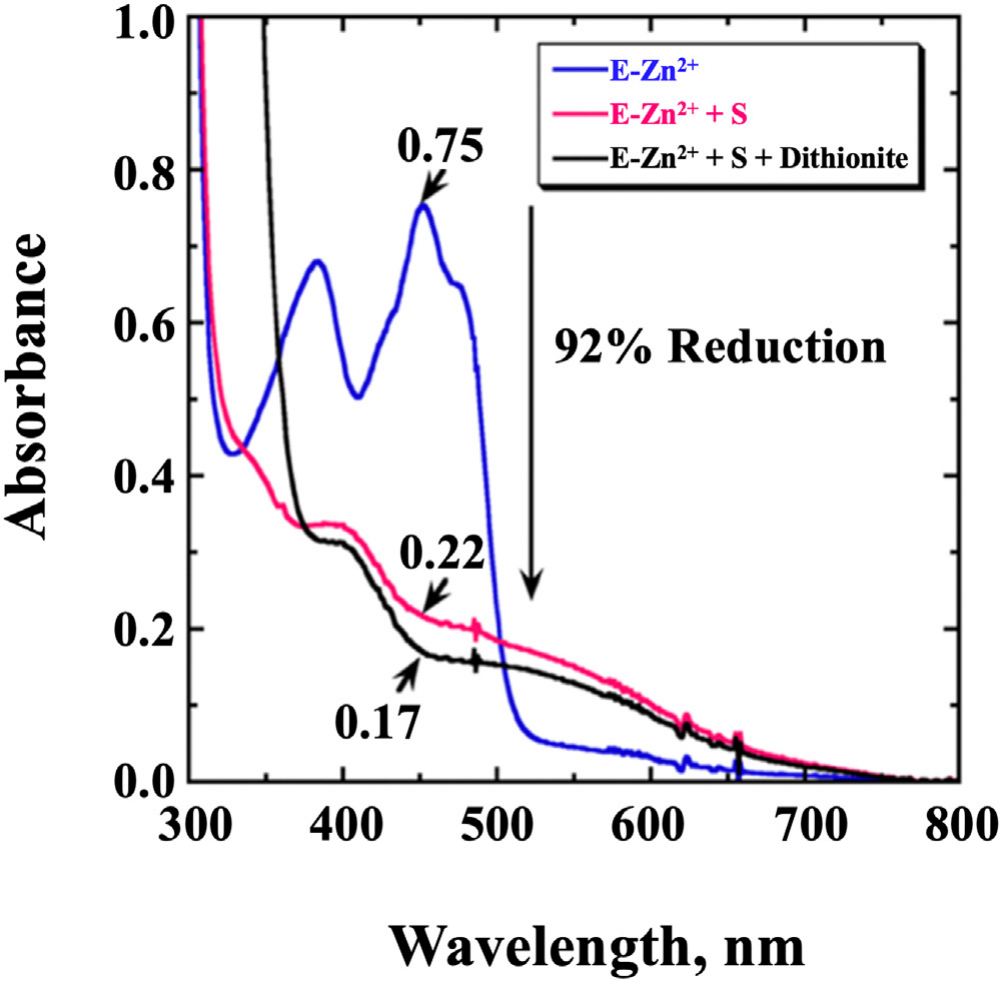
**Substrate-induced reduction of *Pa*D2HGDH after purification with 1 mM Zn**^**2+**^**.** Flavin reduction of *Pa*D2HGDH was recorded for ∼60 μM E-Zn^2+^-bound FAD (*blue*) with 5.4 mM d-malate as substrate in 25 mM NaPO_4_, 1 mM ZnCl_2_, pH 7.4, and 25 °C (*pink*). Sodium dithionite was used as a chemical reducing agent to mark the end point of flavin reduction (*black*). *Pa*D2HGDH, D2HGDH from *Pseudomonas aeruginosa*.

The reduction of the enzyme-bound FAD in E-Zn^2+^ was followed by monitoring the absorbance at 450 nm upon mixing the enzyme with d-malate to investigate whether the enzyme fully loaded with Zn^2+^ could be reduced further than 15%. With 5.4 mM d-malate, there was a 92% reduction of the E-Zn^2+^-bound flavin (Fig. 2). With E-Zn^2+^_100 mM EDTA_ briefly preincubated with 1 mM ZnCl_2_ before the addition of d-malate, a similar 90% flavin reduction was observed (data not shown). In contrast, flavin reduction was only ∼20% when d-malate was added to E-Zn^2+^_100 mM EDTA_ before the addition of ZnCl_2_, suggesting that the order of substrate and metal binding to the enzyme is important.

### Effects of Zn^2+^ on the enzyme-bound flavin

**Figure 3:**
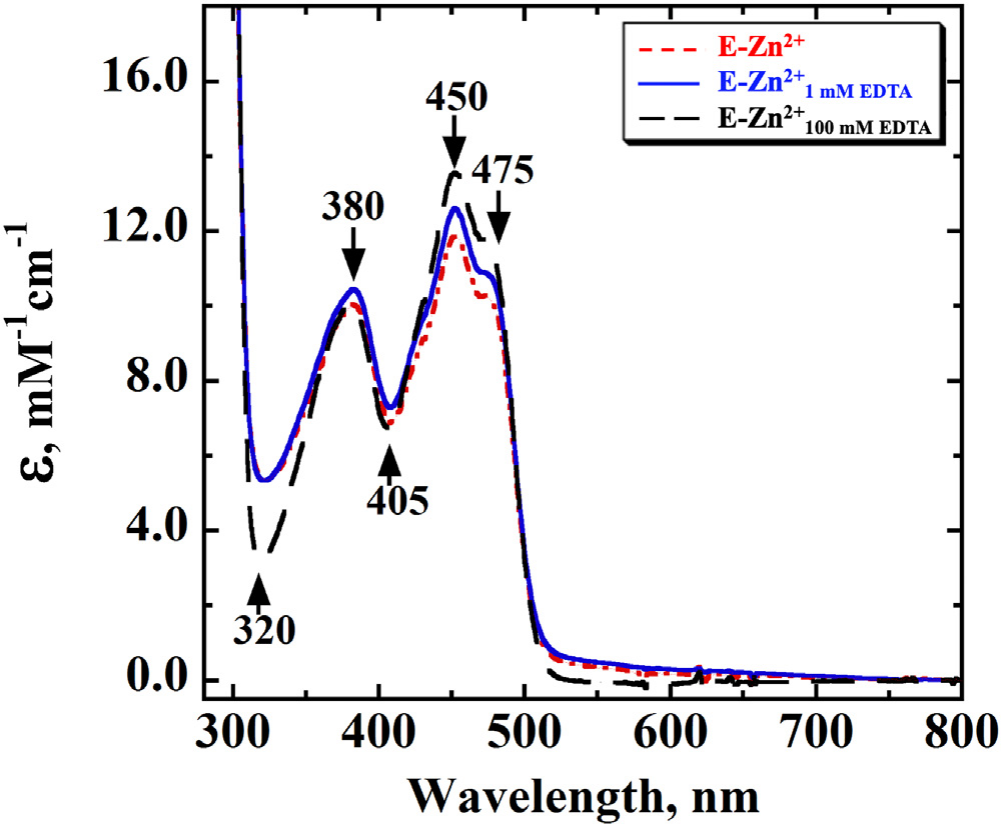
**UV–visible absorption spectrum of *Pa*D2HGDH with and without Zn**^**2+**^**.** The UV−visible absorption spectrum of *Pa*D2HGDH was recorded in 25 mM NaPO_4_ after purification with 1 mM ZnCl_2_ (*red*), treatment with 1 mM EDTA (*blue*), and treatment with 100 mM EDTA (*black*), at pH 7.4 and 25 °C. *Pa*D2HGDH, D2HGDH from *Pseudomonas aeruginosa*.

**Figure 4:**
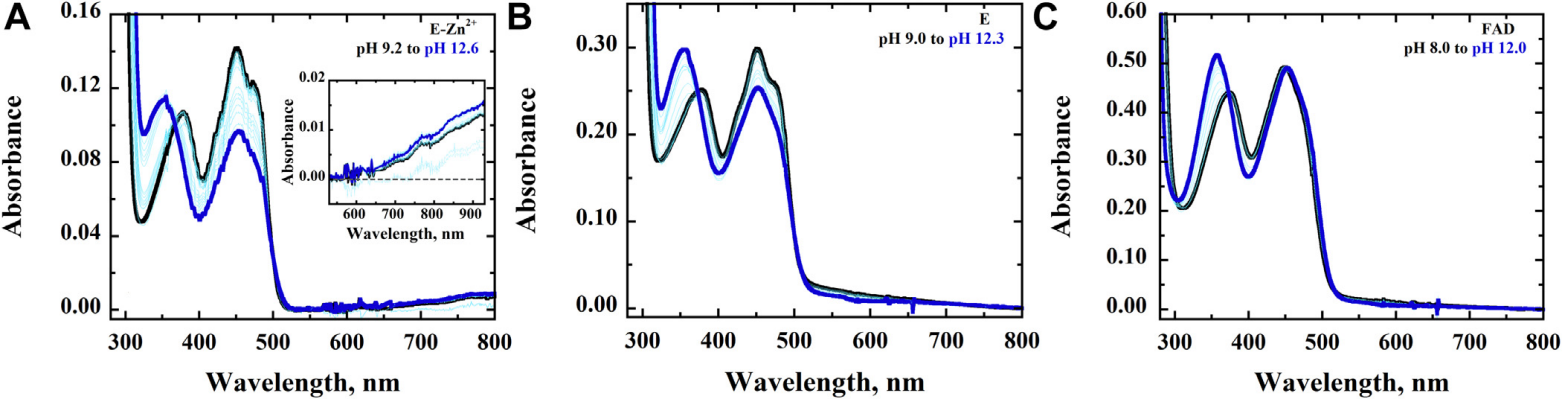
**Effect of pH on the UV–visible absorption spectra of the different enzyme species of *Pa*D2HGDH.** The UV−visible absorption spectra of (*A*) E-Zn^2+^, (*B*) E (E-Zn^2+^_100 mM EDTA_), and (*C*) free FAD were recorded at various pH values adjusted with NaOH in 25 mM NaPO_4_/NaPP_i_ at 18 °C. *A*, *inset*, shows a charge–transfer band around the 530 nm to 930 nm region. *Pa*D2HGDH, D2HGDH from *Pseudomonas aeruginosa*.

**Figure 5:**
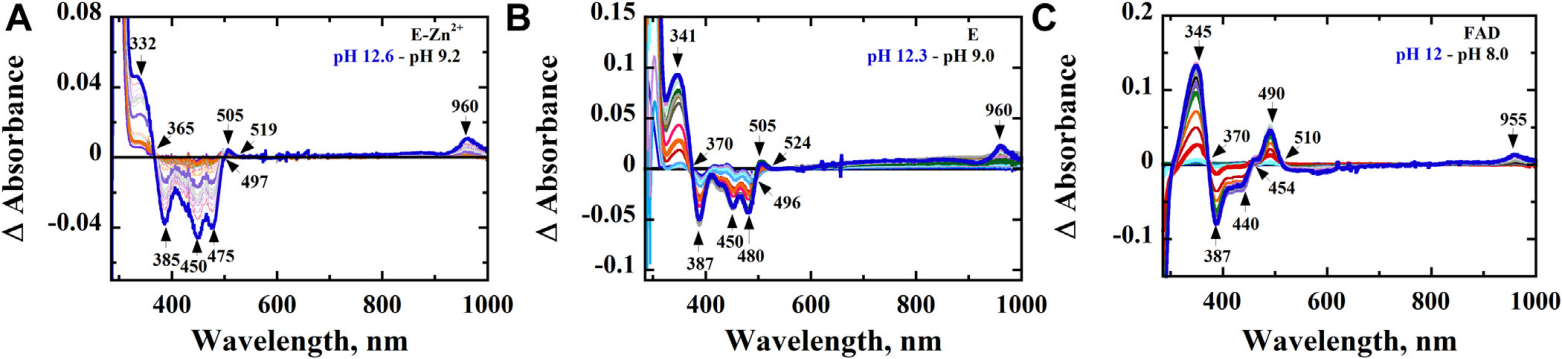
**Difference spectra of the effect of pH on the UV–visible absorption spectra of the different enzyme species of *Pa*D2HGDH.** The UV−visible absorption difference spectra were obtained by subtracting the flavin species at pH ∼9 from all subsequent species for (*A*) E-Zn^2+^; (*B*) E (E-Zn^2+^_100 mM EDTA_), and (*C*) free FAD recorded in 25 mM NaPO_4_/NaPP_i_ at 18 °C. *Pa*D2HGDH, D2HGDH from *Pseudomonas aeruginosa*.

**Figure 6:**
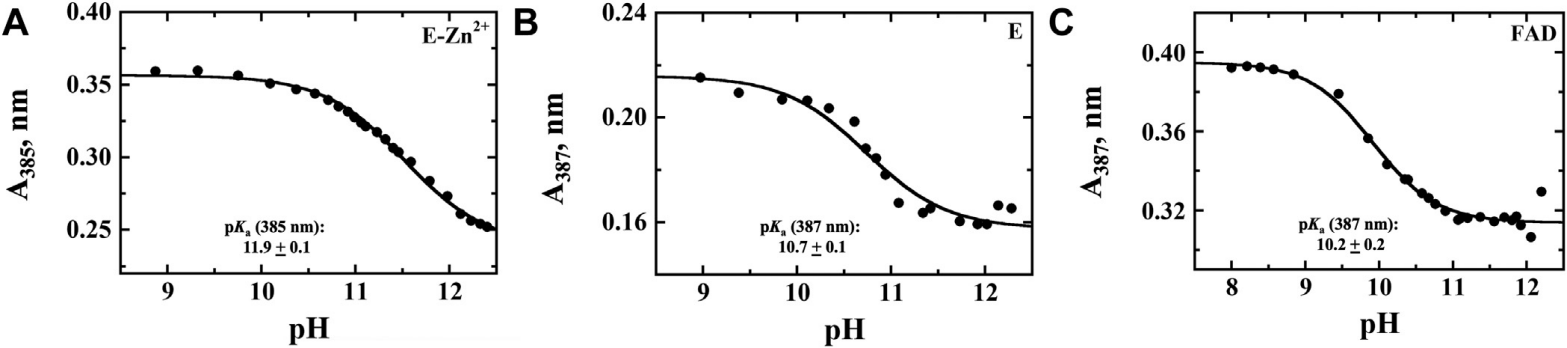
**Plot of the absorbance changes of the flavin high energy valley as a function of pH.** The pH dependence of the high energy valley obtained from the difference spectra after pH titration. *A*, E-Zn^2+^, (*B*) E (E-Zn^2+^_100 mM EDTA_), and (*C*) free FAD after plotting the absorbance values at 387 or 385 nm as a function of pH. The traces and p*K*_a_ values were obtained after fitting the data to Equation 4. The reaction was carried out in 25 mM NaPO_4_/NaPP_i_ at 18 °C.

To elucidate the impact of metal on the spectral properties of the enzyme-bound FAD, the UV−visible absorption properties of E-Zn^2+^, E-Zn^2+^_1 mM EDTA_, and E-Zn^2+^_100 mM EDTA_ were investigated and compared. At pH 7.4, the UV−visible absorption spectra of all three enzyme species showed peaks at 380 and 450 nm, a shoulder at ∼475 nm, and valleys at 320 and 405 nm (Fig. 3). The data are consistent with the reported absorption spectrum of *Pa*D2HGDH purified in the absence of exogenous ZnCl_2_ (44). While the spectra of E-Zn^2+^ and E-Zn^2+^_1 mM EDTA_ generally overlapped, the spectrum of E-Zn^2+^_100 mM EDTA_ showed some differences around the 300 to 450 nm region. To further elucidate whether Zn^2+^ affected the flavin microenvironment of *Pa*D2HGDH, the enzyme-bound flavin for E-Zn^2+^, E-Zn^2+^_1 mM EDTA_, and E-Zn^2+^_100 mM EDTA_ was extracted by heat denaturation and its extinction coefficient when enzyme bound was determined. The resulting flavin ε_450_ values decreased with increasing Zn^2+^ mole ratio as shown in Table 4. To further provide insights on the effect of Zn^2+^ on the electronic properties of the *Pa*D2HGDH-bound flavin, the flavin N^3^ atom p*K*_a_ values of E-Zn^2+^ and E-Zn^2+^_100 mM EDTA_ determined by NaOH titration of the enzyme-bound flavin with simultaneous monitoring of the UV–visible absorption spectra of the flavin species were determined and compared with that of free FAD as shown in Figure 4. The difference spectra for E-Zn^2+^, E-Zn^2+^_100 mM EDTA_, and free FAD generated by subtracting a reference spectrum at low pH from all subsequent spectra are shown in Figure 5. The observed absorbance changes at ∼385 nm as a function of pH were used to determine the p*K*_a_ values of the flavin N^3^ atoms (Fig. 6). The highest p*K*_a_ value was recorded for the flavin N^3^ atom of E-Zn^2+^ (Table 4).

**Table 4:** Effects of Zn^2+^ on the properties of *Pa*D2HGDH-bound FAD

Flavin property	E-Zn^2+^	E-Zn^2+^_1 mM EDTA_	E-Zn^2+^_100 mM EDTA_	Free FAD
ε, M^−1^ cm^−1^^a^	11.8, 10.0 (450, 380 nm)	12.5, 10.4 (450, 380 nm)	13.5, 10.0 (450, 380 nm)	11.3, 9.0 (450, 380 nm)^b^
N^3^ atom’s p*K*_a_	11.9 ± 0.1	NA	10.7 ± 0.1	10.2 ± 0.2

Abbreviation: NA, not available.

a ≤3% standard error was recorded for all numbers.

b Data from Ref. (78); NA, the p*K*_a_ was not determined as E-Zn^2+^ and E-Zn^2+^_1 mM EDTA_ have similar spectral and kinetic properties.

### Kinetics of activation of E-Zn^2+^_100 mM EDTA_ with divalent metals

**Figure 7:**
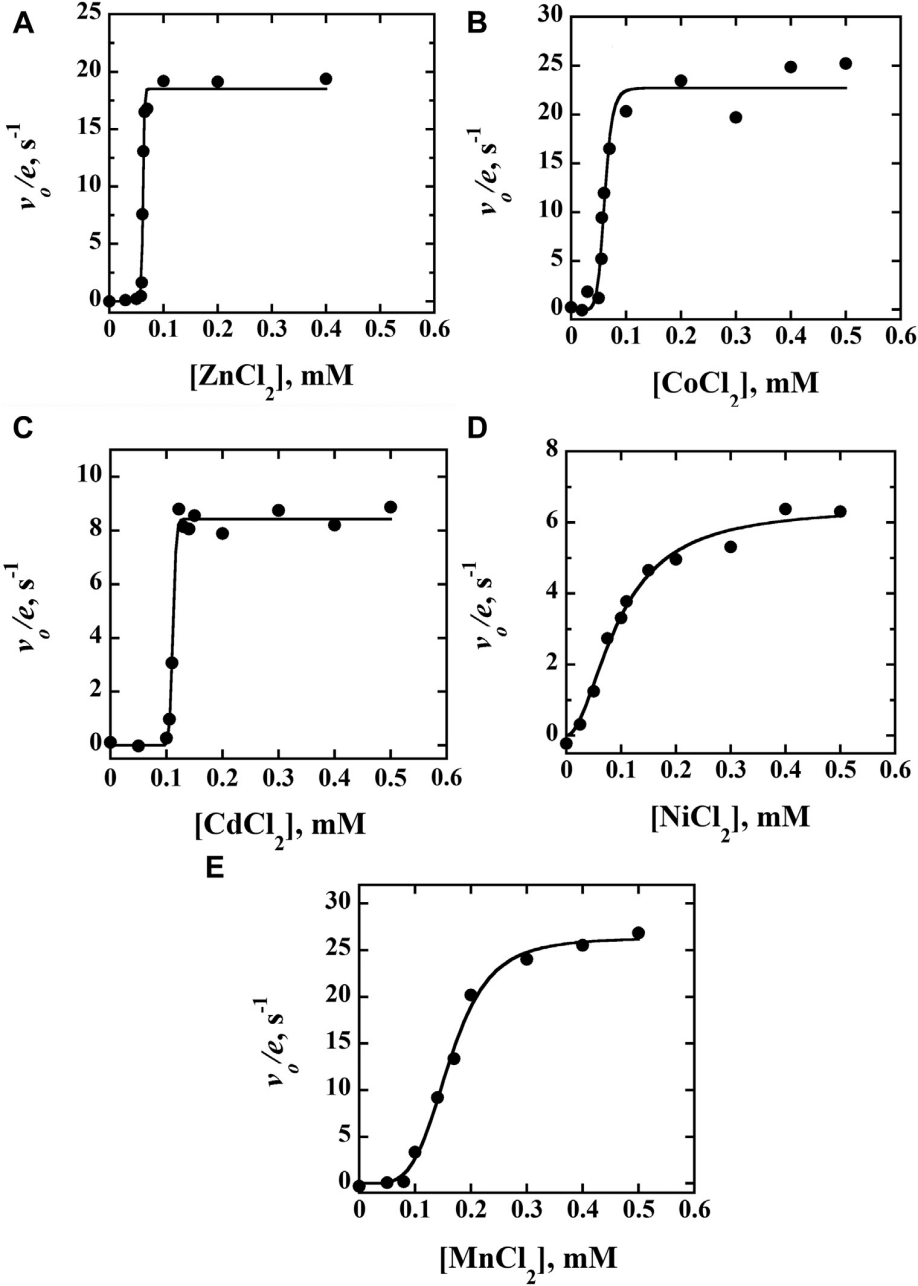
**Metal activation of metallo-apo *Pa*D2HGDH.** Apparent steady-state kinetics of inactivated *Pa*D2HGDH with varying concentrations of chloride salts of activating divalent metals and fixed saturating concentrations of 100 mM d-malate and 1 mM PMS. Enzyme inactivation was obtained after *Pa*D2HGDH treatment with 100 mM EDTA: (*A*) Zn^2+^ activation, (*B*) Co^2+^ activation, (*C*) Cd^2+^ activation, (*D*) Ni^2+^ activation, and (*E*) Mn^2+^ activation. Enzyme activity was measured by monitoring the initial oxygen consumption rates. The data were fit to Equation 5. The reaction was carried out in 25 mM NaPO_4_, 1 mM ZnCl_2_, pH 7.4, and 25 °C. The resulting data extracted from the fit of the plots are summarized in Table 5. *Pa*D2HGDH, D2HGDH from *Pseudomonas aeruginosa*; PMS, phenazine methosulfate.

The activity of E-Zn^2+^_100 mM EDTA_ was determined in the presence of chloride salts of Zn^2+^, Co^2+^, Mn^2+^, Cd^2+^, or Ni^2+^ at pH 7.4 and 25 °C. In all cases, the enzyme activity dependence on the concentration of metal showed a sigmoidal pattern (Fig. 7). From the fit of the data to Equation 5, the concentration of metal yielding a half-maximal increase of enzyme activity, *K*_act_, the maximal and limiting enzyme activity at saturating metal concentration, *k*_lim_, and the activation coefficient, *n*, could be estimated (Table 5). The highest extent of activation, to 19 to 26 s^−1^, was observed in the presence of saturating concentrations of Zn^2+^, Co^2+^, or Mn^2+^ (Fig. 7 and Table 5). In contrast, saturating concentrations of Cd^2+^ or Ni^2+^ yielded increases in the initial rate of reaction to 6 to 8 s^−1^. With Zn^2+^, Cd^2+^, and Co^2+^, the transition from inactive to full enzyme activity was sharp, with *n* values ≥9, and occurred at ∼60 μM with Zn^2+^ and Co^2+^, and ∼110 μM with Cd^2+^. Mn^2+^and Ni^2+^ yielded shallow transitions with *n* values ≤5, with *K*_act_ values of ∼95 μM and ∼160 μM, respectively.

**Table 5:** Activation of E-Zn^2+^_100 mM EDTA_ by divalent metals^a^

Metal (M^2+^)	*k*_*lim*_ (s^−1^)^b^	*K*_*act*_ (μM)^c^	*n*^d^	*R*^2^
Zn^2+^	19 ± 1	62 ± 1	51 ± 8	0.994
Co^2+^	23 ± 1	61 ± 2	9 ± 2	0.982
Ni^2+^	6 ± 1	95 ± 7	2 ± 1	0.994
Cd^2+^	8 ± 1	111 ± 1	41 ± 10	0.993
Mn^2+^	26 ± 1	160 ± 4	5 ± 1	0.998

a Enzyme activity was measured at varying concentrations of metal chloride and fixed saturating concentrations of d-malate and PMS at 100 mM and 1 mM, respectively, in 25 mM NaPO_4_, pH 7.4 and 25 °C, with ∼3 μM protein. The parameters were determined by fitting the data points in Figure 7 to Equation 5.

b *k*_lim_ is the limiting rate of catalysis at saturating metal concentrations.

c *K*_act_ is the metal concentration yielding half *k*_lim_.

d *n* is the activation coefficient associated with the steepness of the sigmoidal activity change in Figure 7.

### Surface electrostatic potential map of *Pa*D2HGDH

**Figure 8:**
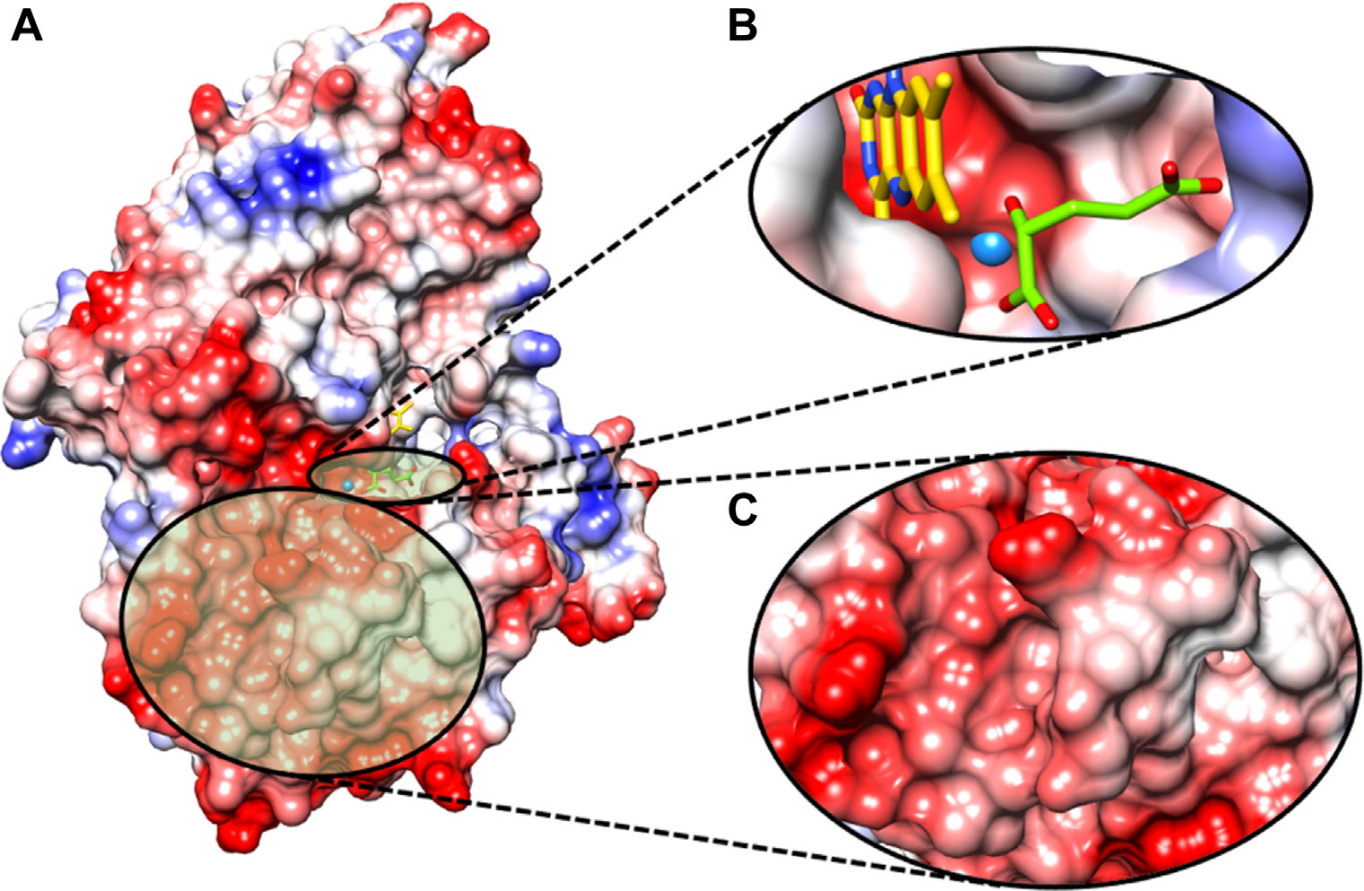
**Electrostatic potential map of *Pa*D2HGDH.***A*, general electrostatic potential map of the *Pa*D2HGDH homology model (44) (built with SWISS-MODEL using a putative dehydrogenase from *Rhodopseudomonas palustris* (Protein Data Bank code: 3PM9). *B*, active site of *Pa*D2HGDH showing electronegative binding pocket of Zn^2+^ (*blue sphere*) with FAD (*yellow sticks*) and D2HG (*green sticks*). *C*, highly electronegative protein surface pockets suitable for nonspecific Zn^2+^ binding to *Pa*D2HGDH. *Red,* electronegative region; *blue*, electropositive region; *white*, neutral region. Images were generated after structural analyses of the *Pa*D2HGDH homology model using UCSF Chimera software (77). *Pa*D2HGDH, D2HGDH from *Pseudomonas aeruginosa*.

To determine possible metal-binding pockets in *Pa*D2HGDH, the electrostatic potential map of the *Pa*D2HGDH homology model was constructed using the UCSF Chimera Coulombic Surface Coloring tool. As seen in Figure 8, an electronegative pocket was identified in the enzyme active site, close to H^374^, H^380^, E^420^, and the N^3^–C^4^O region of the flavin. Analysis of the enzyme's surface revealed multiple regions of solvent-accessible electronegative surface pockets around the active site entrance (Fig. 8).

## Discussion

Kinetic studies, UV–visible absorption analyses, and ICP-MS have been used to investigate the role of metals in *Pa*D2HGDH activity and demonstrate that Zn^2+^ is a cofactor required for the enzyme's activity, rather than an activator. This study also demonstrates that *Pa*D2HGDH can use Co^2+^, Mn^2+^, Cd^2+^, or Ni^2+^ as alternative metals and is inactive when only the flavin cofactor is bound. Evidence to support these conclusions and the kinetic mechanism of metal binding to the metalloapoenzyme are provided later.

### Zn^2+^ is essential for *Pa*D2HGDH activity

This conclusion is drawn from the results of the activity assays and ICP-MS analyses of E-Zn^2+^, E-Zn^2+^_1 mM EDTA,_ and E-Zn^2+^_100 mM EDTA_ (Tables 2 and 3). The data demonstrate that upon treatment of *Pa*D2HGDH with 100 mM EDTA to yield E-Zn^2+^_100 mM EDTA_, the metalloapoenzyme is completely inactive. Thus, Zn^2+^ is an essential cofactor for *Pa*D2HGDH activity. Although Zn^2+^ was found in the active site of human D2HGDH (52) and was reported as an activator for D2HGDHs from other sources and in other FAD-dependent α-hydroxy acid dehydrogenases (25, 26, 27, 28, 29, 30, 31, 32, 33, 34, 35, 36, 37, 38), our study is the first to provide unequivocal evidence of the essentiality of Zn^2+^ for D2HGDHs. Despite other divalent metals providing enzymatic activity to *Pa*D2HGDH, Zn^2+^ was the only one found in significant amounts in the enzyme purified without the addition of any exogenous metal, suggesting that Zn^2+^ is the physiological cofactor for the enzyme. Thus, *Pa*D2HGDH, like the FAD-dependent d-LDHs (25, 43), is a Zn^2+^-dependent metallo flavoenzyme dehydrogenase. Independent evidence that Zn^2+^ is a cofactor for *Pa*D2HGDH activity comes from the substrate reduction of E-Zn^2+^ and the enzyme purified in the absence of ZnCl_2_ (Figs. 1 and 2, Table 2), showing a six fold increase in the yield of flavin reduction in the presence of Zn^2+^. In addition, the ∼90% yield of flavin reduction seen only when the metalloapoenzyme was preincubated with ZnCl_2_ before substrate addition, but not vice versa, suggests that Zn^2+^ binding must precede substrate binding to the metalloapoenzyme for catalysis to occur.

### *Pa*D2HGDH requires a 1:1 ratio of tightly bound Zn^2+^ for activity

This conclusion is supported by the ICP-MS analysis and steady-state kinetics of E-Zn^2+^, E-Zn^2+^_1 mM EDTA,_ and E-Zn^2+^_100 mM EDTA_, as shown in Tables 2 and 3. The *k*_cat_ values reported for E-Zn^2+^ and E-Zn^2+^_1 mM EDTA_ were ∼40 s^−1^, and both enzymes had similar *K*_*m*_ values of ∼4.6 mM for d-malate. The observed similarity in the kinetic parameters of E-Zn^2+^ and E-Zn^2+^_1 mM EDTA_ despite E-Zn^2+^_1 mM EDTA_ having a 1:1 mol Zn^2+^:mol protein ratio demonstrates that only 1 mol of Zn^2+^ is required for maximum activity in 1 mol of *Pa*D2HGDH. Thus, *Pa*D2HGDH is similar to d-LDH from *M. elsdenii*, which contains a Zn^2+^ per FAD (43), but different from F_420_-dependent formate dehydrogenase from *M. formicicum* (34) and FAD-dependent histone demethylase (LSD2) (35), which contain a 2:1 Zn^2+^:protein ratio. The observed 2:1 Zn^2+^:protein ratio for E-Zn^2+^ can be explained as nonspecific loose metal binding to high-density solvent-accessible electronegative surface pockets of *Pa*D2HGDH (Fig. 8). The excess loosely bound metal ions could easily be stripped off by mild concentrations of EDTA, leaving behind the essential and tightly bound Zn^2+^ in the enzyme’s active site as identified through the electrostatic analysis of the *P*aD2HGDH homology model (Fig. 8). This phenomenon has been reported for the binuclear Zn^2+^-binding creatininase from *P. putida* (53). The recently published crystal structure of human D2HGDH provides independent evidence of the presence of an active site Zn^2+^ ion for the enzyme (52).

### H^374^, H^381^, E^420^, and flavin O^4^ most likely coordinate Zn^2+^ binding in *Pa*D2HGDH

**Figure 9:**
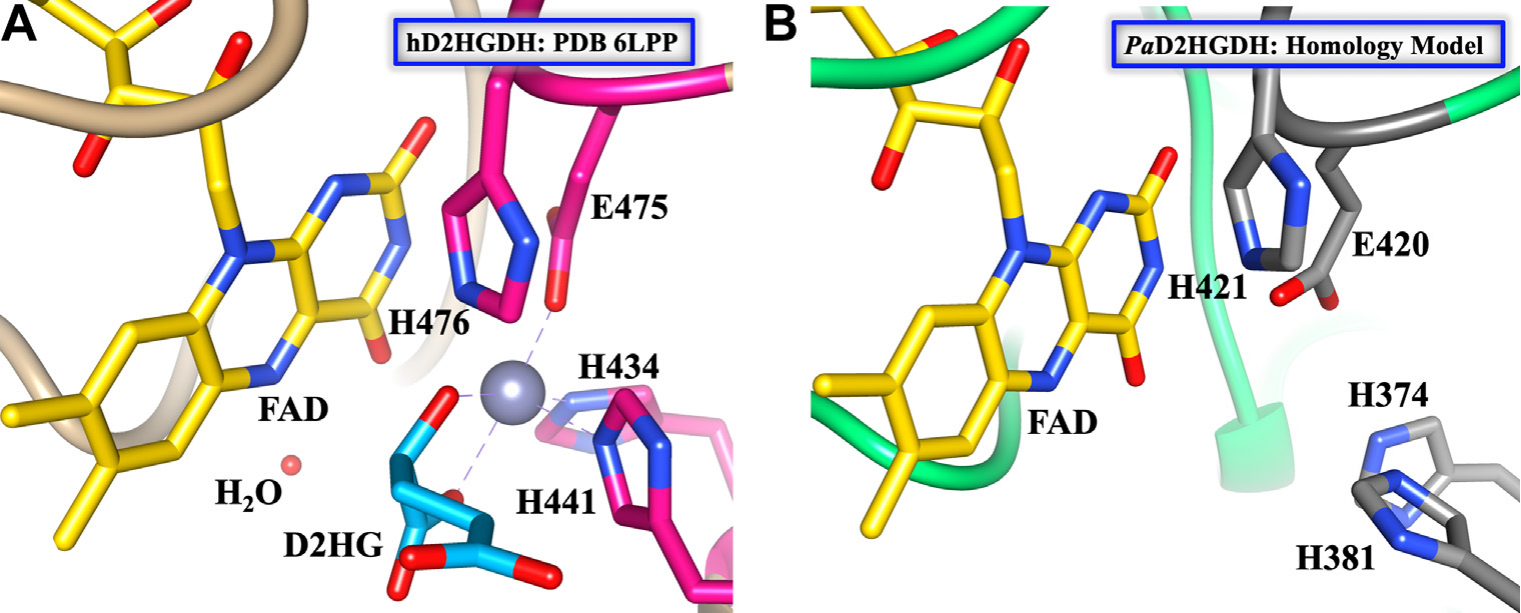
**Proposed residues for Zn**^**2+**^**coordination in *Pa*D2HGDH using human D2HGDH as reference**. *A*, Active-site topology of human D2HGDH (PDB code: 6LPP) (52) with bound D2HG and Zn^2+^ showing residues for Zn^2+^ coordination. *B*, active-site topology of the homology model of *Pa*D2HGDH (44) (built with SWISS-MODEL using a putative dehydrogenase from *Rhodopseudomonas palustris* [PDB code: 3PM9]). Images were generated after structural analyses of the protein files using UCSF Chimera software (77). *Pa*D2HGDH, D2HGDH from *Pseudomonas aeruginosa*.

This conclusion is supported by the identical active-site topologies of the *Pa*D2HGDH homology model (44) and the human D2HGDH crystal structure (52) (Fig. 9). The crystal structure of human D2HGDH reveals a nitrogen–oxygen–nitrogen (N-O-N) Zn^2+^ coordination with active site H^434^, H^441^, E^475^, an active site water molecule, and the flavin O^4^ atom (Fig. 9*A*). Given the full conservation of the *Pa*D2HGDH and human D2HGDH active-site residues (44), and the electrostatic potential map of *Pa*D2HGDH (Fig. 8), an identical Zn^2+^-binding pocket comprising the topologically equivalent active site H^374^, H^381^, E^420^, and the flavin O^4^ atom (Fig. 9*B*) with water/hydroxide coordination in the absence of substrate can be proposed for *Pa*D2HGDH. This N-O-N binding pocket has been described for thermolysin and carboxypeptidase A (24). Thermolysin binds d-Ala–d-Ala through an interaction between Zn^2+^ and the peptide carbonyl group. Thermolysin's transition state involves Zn^2+^ coordination with an oxyanion generated by the nucleophilic addition of water to the peptide carbonyl carbon, resembling the coordination between a carboxylate and an alcohol (54). In the case of carboxypeptidase A, the active site Zn^2+^ coordinates between carbonyl groups and N-H or O-H bonds of peptides or esters (55). By comparison, D2HGDHs bind substrates with similar binding motifs as thermolysin and carboxypeptidase A, that is, a carboxylate and a hydroxyl group, consistent with the proposed N-O-N metal binding pocket of *Pa*D2HGDH. The N-O-N metal binding pocket is not common across alcohol dehydrogenases. Liver alcohol and other alcohol dehydrogenases, which catalyze the oxidation of primary and secondary alcohols (56), coordinate metals using three cysteine residues (S-S-S) and water (57). Notably, other Zn^2+^-dependent enzymes have different binding pockets like the histidine triad (N-N-N) of carbonic anhydrase II and the histidine-cysteine triad (N-N-S) of bacteriophage T7 lysozyme and peptide deformylase (57, 58).

### Zn^2+^ alters the electronic properties of the bound FAD in *Pa*D2HGDH

Evidence to support this conclusion stems from the flavin N^3^ atom deprotonation studies of E-Zn^2+^ and E-Zn^2+^_100 mM EDTA_ and the UV–visible absorption spectra of the different enzyme species (Table 4, Figure 3, Figure 4, Figure 5). The UV–visible absorption spectrum for E-Zn^2+^ showed a decreased intensity of the 450 nm peak with respect to E-Zn^2+^_100 mM EDTA_, with no significant changes around the 380 nm peak and 400 nm valley, consistent with Zn^2+^ hydrate perturbing the electronic environment of the flavin. The observed increase in the flavin N^3^ atom p*K*_a_ value in the presence of Zn^2+^ can be explained by a polarizing effect of Zn^2+^ hydrate on the enzyme-bound flavin. Such a perturbation of the flavin N^3^ atom p*K*_a_ value is likely because of a Zn^2+^-mediated lowering of the p*K*_a_ value of an active-site water molecule coordinating Zn^2+^ to ∼7.0 to form a hydroxide ion, which is the prevalent species coordinated to Zn^2+^ at high pH (57, 59, 60, 61, 62, 63, 64, 65, 66). The proximity of the hydroxide ion's negative charge to the flavin will reduce the likelihood of flavin deprotonation, resulting in an increased flavin N^3^ atom p*K*_a_ value. In addition, the hydroxide ion's proximity to the oxidized flavin results in a charge–transfer complex (67), which is expressed as a long-wavelength absorption of the oxidized flavin above 550 nm, as shown in Figure 4*A*. Independent evidence for the formation of a Zn^2+^-coordinated hydroxide ion in the enzyme's active site comes from pL profiles of the *k*_cat_ and *k*_cat_/*K*_*m*_ values of E-Zn^2+^ with d-malate in aqueous and deuterated buffers, which is presented in the accompanying article (68). The observed decrease in the enzyme-bound flavin ε_450_ value with Zn^2+^ at pH 7.4, on the other hand, is likely because of an electrostatic effect of the metal, because the active-site hydrated Zn^2+^ is only partially neutralized by the coordinated water/hydroxide at low pH. A recent study that investigates the effects of a negative charge on the absorption wavelengths and oscillator strengths of flavins (69) demonstrates an increase in the flavin 450 nm peak intensity. Conversely, a positive charge is expected to decrease the intensity of the 450 nm peak of the flavin, which is expressed as a decreased flavin ε_450_ value upon Zn^2+^ binding to *Pa*D2HGDH. All taken together, the biophysical data presented show that Zn^2+^ alters the electron distribution across the isoalloxazine ring of the enzyme-bound FAD in *Pa*D2HGDH.

### Metal binding to the metallo-apo *Pa*D2HGDH occurs through an induced fit rapid rearrangement mechanism

**Figure 10:**
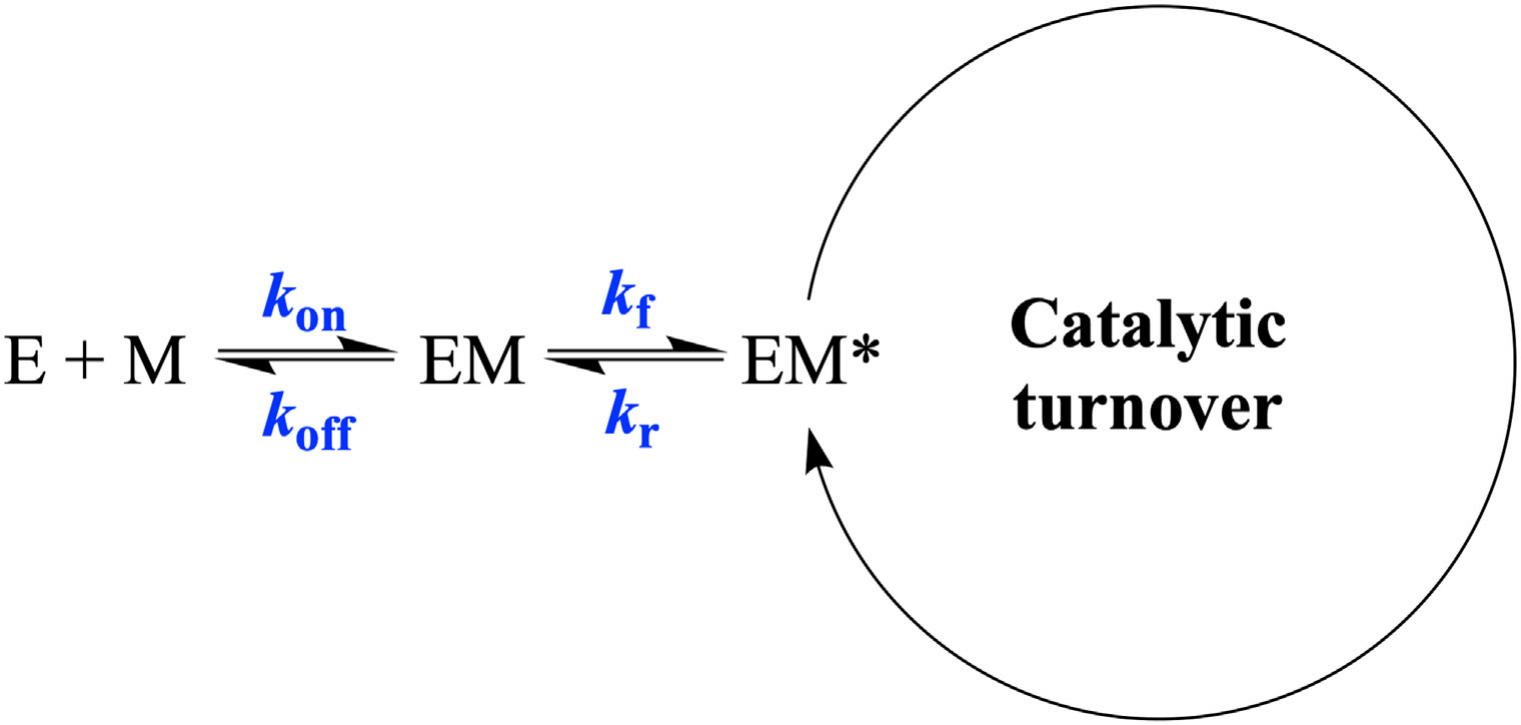
**General kinetic scheme of the induced fit rapid isomerization mechanism**.

Evidence to support this conclusion comes from the plots of the concentration dependence of Zn^2+^, Co^2+^, Mn^2+^, Cd^2+^, or Ni^2+^ on the activity of E-Zn^2+^_100 mM EDTA_ using Equation 1 (Fig. 7 and Table 5). The data plots for all five metals indicate a sigmoidal binding pattern with an abrupt increase in enzyme activity. In a study describing protein–ligand interactions, such a steep-sloped sigmodal pattern has been associated with an induced fit rapid rearrangement model (70). This model describes an enzyme's kinetic behavior that involves two distinct phases: a fast phase and a slow phase. As illustrated in Figure 10, before metal binding, the enzyme exists in the inactive E “off” state. The binding of the metal generates an enzyme–metal EM complex, which undergoes a concentration-dependent rapid conformational change, with the forward and reverse rate constants *k*_*f*_ and *k*_*r*_, respectively, to yield the active EM∗ “on” state. At any given time, the enzyme–metal complex population is split into two states in an equilibrium process. The metal concentration dictates the percentage of the enzyme–metal complex population in the on state or off state. Thus, the relative contributions of the enzyme–metal complex populations in the on and off states, at any given time, create the two distinct slow and fast kinetic phases. The rate constant for metal binding *k*_on_ and dissociation *k*_off_ and the probability of the EM complex to partition forward to yield the EM∗ complex define the slow phase, as shown in Equation 1.

In contrast, the fast phase is given by the sum of the forward and reverse rate constants of enzyme isomerization of the enzyme–metal complexes after metal binding (Equation 2). As the metal concentration increases, the fast phase overcomes the slow phase, and the on state becomes more favored, resulting in the optimal generation of EM∗ that undergoes turnover. The resulting rapid generation of the EM∗ is observed as a sharp rise in enzyme activity (Fig. 7). In Equation 5 and for any given metal, the extent of activation is directly measured from the steepness of the burst phase through the activation coefficient *n* of the sigmoidal plot. A metal that requires the lowest concentration to reach a half-maximal activity, which is probed by the *K*_act_ value, would be the most active. Thus, Zn^2+^ with an *n* value of 50 and a *K*_act_ value of 62 μM is the most preferred metal for *Pa*D2HGDH (Table 5). Of the other four metals restoring the activity of the metalloapoenzyme, Co^2+^ is the only one yielding a *K*_act_ value of 61 μM, which is comparable to that seen with Zn^2+^. In addition, the activity of the metalloapoenzyme increased to a *k*_lim_ value of ∼20 s^−1^ for the maximal enzyme activity at saturating metal concentration with both Zn^2+^ and Co^2+^, suggesting that the two metals play an interchangeable role in the enzyme's function. An increase in the enzyme's activity to a similar extent by Zn^2+^ and Co^2+^ was previously demonstrated in carbonic anhydrase (66). Similar sigmoidal patterns have been reported for the activation, that is, opening, of gated ion channels in response to metals and tissue responses to irreversible electroporation during tumor suppression (71, 72, 73, 74).

(1) $$k_{slow}=k_{on}\;\left[l\right]+k_{off}\left(\;\frac{k_{r}}{k_{f}+k_{r}}\;\right)$$

(2) $$k_{fast}=k_{f}+k_{r}$$

In conclusion, this study establishes that *Pa*D2HGDH is a metallo flavoenzyme dehydrogenase that requires an essential and tightly bound Zn^2+^ for activity with a metal:protein ratio of 1:1. The enzyme is inactivated upon removal of the bound Zn^2+^ and is restored in its kinetic properties when exogenous amounts of the chloride salts of Co^2+^, Zn^2+^, Mn^2+^, Cd^2+^, or Ni^2+^ are added. The binding of the divalent metals to *Pa*D2HGDH occurs through an induced-fit rapid rearrangement mechanism. For most alcohol dehydrogenases, such as liver alcohol dehydrogenase (56, 57), that employ metals as cofactors, the alcohol substrates oxidized during catalysis are usually simple alcohols, such as butanol. In the case of alcohol dehydrogenases that utilize flavins as cofactors, the alcohol substrates oxidized are generally primary and secondary alcohols such as isopropanol (75). In NADH-dependent α-hydroxy acid oxidases such as the *Plasmodium vivax* LDH, the enzyme's active site is designed with positively charged amino acid residues to coordinate the lactate carboxylate group (76). In *Pa*D2HGDH, nature employs a metal besides a flavin for catalysis to yield a neutral active site for the oxidation of the negatively charged dicarboxylic acid substrate. The study also demonstrates that Zn^2+^ alters the electronic properties of the bound flavin through a metal-mediated polarizing effect that results in altered UV–visible absorption spectra, increased flavin N^3^ atom p*K*_a_ value, and decreased ε_450_ value of the flavin with Zn^2+^. This study provides a remedy for the incomplete flavin reduction observed in D2HGDHs and a rationale for the need for two cofactors, that is, a flavin and a divalent metal. The study also demonstrates mechanisms of *Pa*D2HGDH activation and inactivation that can serve as a direction for future designs of new therapeutic targets against *P. aeruginosa* infections.

## Experimental procedures

### Materials

The *Pa*D2HGDH pET20b(+) plasmid harboring the PA0317 gene was designed in-lab and purchased from GenScript. The plasmid was sequenced to verify the presence of the wildtype gene. *E. coli* strain Rosetta(DE3)pLysS was from Novagen. Bovine serum albumin was purchased from Promega. Luria–Bertani (LB) agar, LB broth, chloramphenicol, IPTG, lysozyme, sodium hydrosulfite (dithionite), PMS, and PMSF were obtained from Sigma–Aldrich. Ampicillin was purchased from ICN Biomedicals. d-2-hydroxyglutarate was purchased from MilliporeSigma. d-malate was purchased from Alfa Aesar. EDTA, glycerol, and all other reagents were of the highest purity commercially available.

### Expression and purification of *Pa*D2HGDH

To obtain pure enzyme for kinetic studies, a 10 ml LB broth medium containing 100 μg/ml ampicillin and 34 μg/ml chloramphenicol was inoculated with frozen stocks of *E. coli* cells Rosetta(DE3)pLysS harboring the *Pa*D2HGDH pET 20b(+) plasmid. The cell cultures were used to inoculate 1 l LB broth and incubated on a rotatory plate at 37 °C and 180 rpm for 18 h. Protein expression was induced with 100 μM IPTG when cell density reached an absorbance at 600 nm of ∼0.6. The temperature of the culture was then lowered to 18 °C while shaking on a rotatory plate at 180 rpm. After 17 h of expression, the cells were harvested by centrifugation for 30 min at 2800*g* and 4 °C.

The lysis buffer containing 1 mM PMSF, 2 μg/ml DNase or RNase, 4 mg/ml lysozyme, 5 mM MgCl_2_, 300 mM NaCl, 10 mM imidazole, 10% glycerol, and 20 mM NaPO_4_, pH 7.4, was used to resuspend the wet cell paste in a ratio of 1 g of the wet cell paste to 4 ml of lysis buffer. The suspended cells were then incubated for 30 min on ice while stirring. The resulting slurry was sonicated in five cycles of 5 min each with 5 min off intervals, and then the cell debris was removed by centrifugation at 11,200*g* for 30 min. The resulting cell-free extract supernatant was purified to homogeneity using a nickel–nitrilotriacetic acid column, equilibrated with buffer A (20 mM Tris–Cl, 10 mM imidazole, 300 mM NaCl, and 10% glycerol, pH 7.4). The purification was carried out using a Unicorn ÄKTA Start purification system. Elution of the bound protein was through a gradient from 0 to 100% buffer B (20 mM Tris–Cl, 500 mM imidazole, 300 mM NaCl, and 10% glycerol, pH 7.4), with *Pa*D2HGDH eluting at ∼40% buffer B. The solution containing the purified protein, typically 15 ml, was dialyzed against five 2 l changes of 10% glycerol, 20 mM Tris–Cl, pH 7.4, for 24 h, at 4 °C. The purified enzyme was stored in single-use aliquots in 10% glycerol, 20 mM Tris–Cl, pH 7.4, at −20 °C, and was stable for at least 6 months.

To obtain the Zn^2+^-bound enzyme, purifications were carried out by following the aforementioned protocol using 25 mM NaPO_4_ as a buffer with 1 mM ZnCl_2_ in all purification buffers.

To rid the enzyme of all bound metals, dialysis of the purified enzyme was performed in 2 l volumes of 100 mM NaPO_4_, 1 or 100 mM EDTA, 1 mM dithiothreitol, pH 7.4, for 72 h with no buffer changes at 4 °C.

### Reductive-half reaction

To determine the *K*_*d*_ values for D2HG and d-malate with the recombinantly expressed *Pa*D2HGDH without Zn^2+^, the reduction of the enzyme-bound flavin was followed by monitoring the decrease in absorbance at 450 nm upon mixing *Pa*D2HGDH anaerobically with varying concentrations of the reducing substrate. The time-resolved absorption spectroscopy of the reduction of the enzyme-bound flavin with D2HG or d-malate was carried out with an SF-61DX2 Hi-Tech KinetAsyst high-performance stopped-flow spectrophotometer (TgK-Scientific) thermostated at 25 °C and equipped with a photomultiplier detector under anaerobic conditions. The reductive half-reaction was performed under pseudo–first-order conditions where the enzyme concentration after mixing with the substrate was ∼9 μM, and that of the reducing substrate was between 80 and 800 μM for D2HG or 0.6 and 60 mM for d-malate. The enzyme purified without Zn^2+^ was equilibrated with 25 mM Tris–Cl, pH 7.4, using a PD10 column. Equal volumes of the enzyme and the reducing substrate were mixed in the stopped-flow spectrophotometer in single-mixing mode following established procedures. The instrument dead time for mixing was 2.2 ms.

The stopped-flow traces were fit to Equation 3 using the KinetAsyst 3 (TgK-Scientific) software, which describes a double-exponential process where *A* represents the absorbance at 450 nm at time *t*, *B*_*1*_, and *B*_*2*_ represent the amplitudes of the decrease in absorbance, *k*_obs1_ and *k*_obs2_ define the observed rate constants for the change in absorbance associated with flavin reduction. *C* is an offset value accounting for the nonzero absorbance of the enzyme-bound reduced flavin at an infinite time.

(3) $$A={B_{1}}^{{-k}_{obs1}t}+{B_{2}}^{{-k}_{obs2}t}+C$$

### *Pa*D2HGDH activity with divalent cations

To investigate the effect of divalent cations on the activity of natively expressed *Pa*D2HGDH purified without metals, the enzyme's activity was analyzed by adding exogenous amounts of 100 μM chloride salts of Co^2+^, Ca^2+^, Zn^2+^, Mn^2+^, Mg^2+^, Fe^2+^, Cd^2+^, Cu^2+^, or Ni^2+^ to an enzyme reaction mixture. The enzyme reaction mixture comprised of buffer, *Pa*D2HGDH, reducing substrate, and an artificial electron acceptor. Activity assays for *Pa*D2HGDH with 5 mM d-malate as a substrate and 1 mM PMS as the electron acceptor were measured by monitoring the initial oxygen consumption rates with a computer-interfaced Oxy-32 oxygen-monitoring system (Hansatech Instruments Ltd) after the exogenous addition of metals in 25 mM Tris–Cl, pH 7.4 and 25 °C. The data were analyzed using Microsoft Excel (data not shown).

### *Pa*D2HGDH metal component analysis

To determine the concentrations of divalent metals incorporated into *Pa*D2HGDH after recombinant expression in *E. coli* Rosetta(DE3)pLysS cells after purification with or without the exogenous addition of Zn^2+^, the metal composition of *Pa*D2HGDH was investigated using ICP-MS analysis. Aliquots of all enzyme species, namely the enzyme purified without exogenous Zn^2+^ (E), the enzyme purified and stored in NaPO_4_ and ZnCl_2_ (E-Zn^2+^), the enzyme treated with 1 mM EDTA (E-Zn^2+^_1 mM EDTA_), and the enzyme treated with 100 mM EDTA (E-Zn^2+^_100 mM EDTA_), were dialyzed against 2 l of deionized water for 24 h. The resulting enzyme solutions were then submitted for ICP-MS analyses at the Center for Applied Isotope Studies, University of Georgia, Athens, GA. The resulting data were analyzed using Microsoft Excel.

### Oxygen reactivity

To investigate the effect of Zn^2+^ incorporation on the O_2_ reactivity of *Pa*D2HGDH, the enzymatic activity of E-Zn^2+^ with molecular oxygen was measured by monitoring the initial oxygen consumption rate with a computer-interfaced Oxy-32 oxygen-monitoring system (Hansatech Instruments Ltd) at 25 °C. To test for oxygen reactivity, the reaction mixture contained 40 nM enzyme and d-malate between 1.6 and 40 mM in 25 mM NaPO_4_, pH 7.4. After ∼3 min of reaction time, 1 mM PMS was added to the reaction mixture to regenerate the oxidized state of the enzyme at the expense of O_2_. The instantaneous reoxidation of the enzymatically reduced PMS by molecular oxygen was then observed (data not shown).

### Enzyme activity assay

To investigate the kinetic properties of the various *Pa*D2HGDH species, the apparent steady-state kinetic parameters of E-Zn^2+^, E-Zn^2+^_1 mM EDTA_, and E-Zn^2+^_100 mM EDTA_ with d-malate as a substrate and PMS as an artificial electron acceptor were determined by monitoring the initial rates of oxygen consumption with a computer-interfaced Oxy-32 oxygen-monitoring system (Hansatech Instruments Ltd). d-malate concentrations were between 1.6 and 40 mM, and PMS concentration was fixed at a saturating concentration of 1 mM. The enzyme concentration was 7 nM, and the buffer was 25 mM NaPO_4_, pH 7.4, and 25 °C.

Data analysis was performed using the KaleidaGraph software (Synergy Software) or Enzfitter software (Biosoft). For the apparent steady-state kinetics, the Michaelis–Menten equation was used.

### Substrate-induced reduction of E-Zn^2+^

To determine the effect of Zn^2+^ incorporation on the reductive properties of the enzyme-bound FAD, the percent flavin reduction of E-Zn^2+^ in the presence of d-malate was investigated. The reaction followed the UV−visible absorption peak of E-Zn^2+^ oxidized flavin at 450 nm in a 1 cm path length quartz cuvette using an Agilent Technologies model HP 8453 PC diode-array spectrophotometer equipped with a thermostated water bath. The experiment was carried out under anaerobic conditions to avoid any potential effects of O_2_ on flavin reduction. The enzyme was initially incubated in 25 mM NaPO_4_, pH 7.4, followed by the repeated addition of d-malate aliquots until a final d-malate concentration of ∼5.4 mM was achieved. The quenching of the 450 nm peak associated with flavin reduction was observed with each addition of d-malate. To determine the end point of flavin reduction, sodium dithionite was added to the reaction mixture as a chemical reducing agent. The absorbance at 450 nm after adding sodium dithionite was marked as the endpoint of flavin reduction. The spectra data were analyzed using the KaleidaGraph software.

### Effects of Zn^2+^ on the biophysical properties of *Pa*D2HGDH-bound FAD

To understand the impact of Zn^2+^ on the spectral properties of *Pa*D2HGDH, the various enzyme species were subjected to UV−visible absorption studies. The spectral properties of E-Zn^2+^, E-Zn^2+^_1 mM EDTA_, and E-Zn^2+^_100 mM EDTA_ were investigated using an Agilent Technologies model HP 8453 PC diode-array spectrophotometer equipped with a thermostated water bath at 18 °C. The spectroscopic fingerprints for all enzyme species were determined by obtaining the absorption spectra of the enzyme-bound flavin between 280 and 800 nm. The flavin absorbance data were observed using a 1 cm path-length quartz cuvette. The spectra data were analyzed using the KaleidaGraph software.

To understand the impact of Zn^2+^ on the flavin microenvironment of *Pa*D2HGDH, the extinction coefficient of the enzyme-bound FAD was determined. The extinction coefficients of E-Zn^2+^, E-Zn^2+^_1 mM EDTA_, and E-Zn^2+^_100 mM EDTA_ were determined after enzyme heat denaturation at 100 °C for 20 to 40 min. The absorbance values of the extracted flavins followed at 450 nm were used to calculate the molar extinction coefficients for the respective enzyme species. The extinction coefficient of free FAD at 450 nm was used as the reference in determining the molar extinction of the FAD bound to E-Zn^2+^, E-Zn^2+^_1 mM EDTA_, or E-Zn^2+^_100 mM EDTA_.

To understand the impact of Zn^2+^ on the electronic properties of the *Pa*D2HGDH-bound flavin, the p*K*_a_ values of the flavin N^3^ atoms of E-Zn^2+^ and E-Zn^2+^_100 mM EDTA_ were investigated and compared with that of free FAD. E-Zn^2+^_1 mM EDTA_ was not investigated in this analysis because the enzyme is expected to behave like E-Zn^2+^ after observing similar spectral and kinetic properties for E-Zn^2+^_1 mM EDTA_ and E-Zn^2+^ (*vide supra*). The p*K*_a_ values of the flavin N^3^ atoms of E-Zn^2+^ and E-Zn^2+^_100 mM EDTA_ were investigated by NaOH titration of the enzyme-bound flavin with simultaneous monitoring of the UV–visible absorption spectra of the flavin species from pH 9.0 to 12.5. The enzymes were stable at all pH values tested. The resulting spectra were then analyzed using the KaleidaGraph software. Flavin difference spectra were generated by subtracting the spectrum of a reference pH, that is, 9.0 for E-Zn^2+^ and E-Zn^2+^_100 mM EDTA_ and 8.0 for free FAD, from all subsequent spectra. The changes in the absorbance of the high energy valley for each flavin species at 385 nm for E-Zn^2+^ and 387 nm for E-Zn^2+^ and free FAD were plotted as a function of pH. The resulting plots were fit to Equation 4, which describes the sigmoidal decay of the absorbance at ∼385 nm as a function of pH. *Y* is the absorbance at ∼385 nm. *C*_*L*_ and *C*_*H*_ represent low and high limiting values describing lower and upper offsets in absorbance changes. p*K*_a_ is the resulting p*K*_a_ value of the flavin N^3^ atom. The spectra data were analyzed using the KaleidaGraph software.

(4) $$Y=\frac{C_{L}+C_{H}\;\left(10^{\frac{pK_{a}}{pH}}\right)}{1+10^{\frac{pK_{a}}{pH}}}$$

### Reactivation of inactive *Pa*D2HGDH by metals

To investigate the effects of divalent metals on *Pa*D2HGDH activity, the concentration dependence of Co^2+^, Zn^2+^, Mn^2+^, Cd^2+^, or Ni^2+^ on the activity of E-Zn^2+^_100 mM EDTA_ was investigated in 25 mM NaPO_4_, pH 7.4, at 25 °C, with fixed concentrations of d-malate and PMS at 100 mM and 1 mM, respectively. The initial enzyme reaction rates were monitored after adding exogenous chloride salts or the metals to yield final concentrations between 0.01 mM and 0.5 mM. The resulting data were fit to Equation 5, in which *Y* is the fraction of *Pa*D2HGDH concentration bound by the metal, [*M*] is the total metal concentration, *n* is the activation coefficient, *K*_act_ is the metal concentration producing half-saturation of *Pa*D2HGDH, and *k*_lim_ is the maximal and limiting enzyme activity at saturating metal concentration. The data were analyzed using the KaleidaGraph software.

(Eq 5) $$Y=\frac{k_{lim}{\left[M\right]}^{n}}{K_{{act}^{n}}+{\left[M\right]}^{n}}$$

### Electrostatic potential map of *Pa*D2HGDH

To determine possible metal-binding pockets in *Pa*D2HGDH, the electrostatic properties of the *Pa*D2HGDH homology model (44) were investigated using the UCSF Chimera software (77). The *Pa*D2HGDH electrostatic potential revealing the surface and binding properties of the protein was generated using the UCSF Chimera Coulombic Surface Coloring tool, which calculates the electrostatic potential according to Coulomb’s law. The color code was set to present red for negative potential, white for neutral, and blue for positive potential. The resulting protein surface and active-site potential maps were then saved as images for graphical comparison. To better understand the role of enzyme electrostatic potential in the binding of ligands to *Pa*D2HGDH, the physiological substrate D2HGD and Zn^2+^ were modeled into the *Pa*D2HGDH homology model and analyzed in light of the electrostatic potential map generated for the enzyme’s active site.

## Data availability

All data are contained within the article.

## Conflict of interest

The authors declare that they have no conflicts of interest with the contents of this article.
